# What Shall We Do about Porcelain

**Published:** 1903-12-15

**Authors:** Edgar A. Honey

**Affiliations:** Kalamazoo, Mich.


THE
### DENTAL REGISTER.

**Vol. LVII. DECEMBER 15, 1903. No. 12.**

### WHAT SHALL WE DO ABOUT PORCELAIN.

BY EDGAR A. HONEY, D.D.S., KALAMAZOO, MICH.

Read before the Central Michigan Dental Association, November 4, 1903.

A great deal has been written during the past few years regarding the use of porcelain as a filling material, and it is this phase of the subject that I wish to discuss with you for a few moments, and I promise to be short.

I will state at the outset that I have no new principles to advance, and no new methods in the manner of working porcelain; but rather to criticise kindly the attitude that many of the best men in the profession have assumed and are assuming toward this very important branch of our work.

How often do we hear some good skillful dentist say, "Oh! I am too busy to 'monkey' with porcelain," or another will say, "I do a little porcelain work when I'm not too busy." A member of the faculty of one of our prominent dental colleges said to me a few months ago, "Oh! porcelain is all very well as a plaything, but a busy dentist has no time for it."

Now, it is to this busy dentist, who has not yet taken the initial step in inlay-work, that I wish to address my remarks, and if I should succeed in awakening the interest in some one of you for the work I am to discuss, I shall feel well repaid for my visit to this meeting.

The question with the busy dentist, or any other dentist, should not be how much time is consumed, but what use, in its broadest sense, is porcelain as a filling material and in what degree can we better serve our patients by its use than the use of the materials ordinarily placed in our hands for this work.

Many of us, though thoroughly convinced of the merit from every standpoint, put off from week to week our beginning in porcelain from sheer procrastination—very much in the position of the man who hesitates to take a cold plunge in the morning, he knows the results are good, but dreads the shock resulting from so sudden a change of temperature. In the same manner we dread our first few failures and rather begrudge the time spent in hard work that fails to make a creditable showing on our day-book. I ask in all sincerity if this is not so in many cases?

If I may be permitted at this point, I will relate my own experience in this respect. I will say that I had a Hammond furnace in my office for fully a year before I made my first inlay, waiting patiently for the time when I had leisure moments that might be improved by taking the first steps in porcelain work.

We all remember how slow, tedious and exhausting were our first operations in college when we attempted to manipulate gold. One fair-sized filling consumed most of the time of half a day's session, yet this very work and patience was necessary to overcome the difficulties attendant upon this work, and why should we expect to jump into porcelain work at one bound and make our time as profitable to us as it would be were we manipulating a material with which we were perfectly familiar.

The busier a dentist is the greater is the responsibility resting on his shoulders, that he give his patients the very best service in each individual case that the immediate conditions demand.

If we wish to know what may be expected of porcelain as a filling material, the majority of us are obliged to rely upon the experiences of those who may be called for the time being the pioneers in this work. In this connection I will quote two or three such.

Regarding porcelain as a permanent filling material, Dr. Reeves, to whom by the way all porcelain workers cheerfully raise their hat, has this to say: "It is an unaccountable fact by the clinical experiences of all inlay workers of any considerable period of time that there is seldom or never any recurrence of decay around inlays. In a period extending over ten years, I have yet to have the first case of recurrence of decay around an inlay." This last statement, coming from one whose sincerity and modesty is unquestioned, is really a wonderful testimony in favor of porcelain as a permanent filling material.

Let us now see what some others may have to offer along the same line. In *Items of Interest* of January, 1902, is an article entitled "Porcelain filling after twelve years," by W. S. Capon, of Philadelphia. In the course of his remark Dr. Capon has this to say: "It is a surprise to know the great durability of corner sections. I have a number of cases ranging from eight to twelve years of service, that I have watched with great interest, and in a few cases they have been positively abused, and yet they stand the strain although held with cement, without the aid of pins or other means." Quantity of testimony, equally convincing as that quoted, might be brought to your notice, but this is sufficient, although we know nothing farther, either in our own experience or those of our immediate acquaintances to convince us that it should receive our careful attention, and that we should undertake a thorough study of the principles involved and the methods necessary to success.

One of the easiest things to dispense, and of which the most of us have an unlimited supply, is advice. And yet in the face of this fact, I will be bold enough to offer a word of advice, based upon my own experience, to the busy dentist who has recognized the importance of porcelain, and, through lack of time, is unable to devote many hours to experimental work on models, etc. After you have given the subject thorough and careful study, as to how the simplest cavities should be prepared for inlays, select the first favorable case that comes to your hands and give up the entire time reserved for one sitting, if need be, in the preparation of the cavity for an inlay, being careful not to slight the merest detail. If your cavity has been properly prepared, there is little doubt that the balance of the work, aside perhaps from getting the exact shade, will be accomplished in a manner to obtain a gratifying measure of success, even in the case of your first work. If the time reserved for this patient has been consumed, let the matrix go over to the next sitting and when that time arrives devote the entire sitting to making a matrix and if time will allow make two or three. The baking of the porcelain may be done at odd times between sittings, providing you have decided on the colors to be used.

When you come to set your inlay, you will be surprised to see how well you have succeeded in your first attempt, providing you have clearly in your mind how you are going to take each step and have not attempted to accomplish too much in a short time. To those of you who are provided with two chairs I would say set your inlay according to the directions so carefully laid down by Dr. Reeves. Put your patient in the second chair allowing rubber dam to remain in place for a half hour while you may be giving your attention to the next patient. I am thoroughly convinced that the busy dentist will profit infinitely more by doing his very first work on patients and not on models. I am so convinced of this, that I wish you to understand that I place special emphasis on this point. Devote plenty of time to your first few cases and as much time at each sitting as circumstances will permit. You will be surprised to see how soon you will begin to gain confidence in yourself, and the appreciations shown by your patients will amply repay you for all the effort it has cost.

I made my first inlay a few months ago out of what seemed to be sheer necessity. A young patient presented with the mesial third of a central incisor broken off. Three gold fillings had failed successively all within a few months, each time breaking an additional portion of tooth structure. The adjoining teeth were sound and beautiful, and the disfigurement that must follow the insertion of a gold filling so impressed me, that I asked her to wait a week or so, in the meantime I would decide how best to restore the broken corner.

I canceled my work for an entire afternoon and went to work. I completed the inlay and attempted to set it but used too much pressure laterally and scaled off a layer of porcelain. Two days later I made another to replace the broken one, in the meantime modifying the shape of my cavity, providing a more definitely-outlined seat for the inlay.

Since that time or during the past five months I have inserted something over eighty inlays. Of these three have come out, due to faulty cavity preparation. Two or three others I am going to replace. From the majority of the balance I look for a fair degree of successful service. I keep a special record of my inlays and will examine them by appointment at stated times and record the condition I find each time. In this way I hope to keep close watch of my failures and perhaps learn by hard experience a few of the many things regarding porcelain work that many of the experts have been attempting to teach us through the dental journals during the past two or three years.

So far, I have said nothing about the two factions, so to speak, namely the advocates of high or low-fusing bodies,

Regarding the low-fusing body or the gold matrix, Dr. Jenkins, of Dresden, probably its greatest exponent, in a paper read before one of European dental societies, makes the statement that he belives that no one within the sound of his voice would think of using a material so intractable as platinum for obtaining a matrix.

To this Dr. Reeves replies in a paper read before the Iowa State Dental Society, 1902, referring to the Jenkins system, he says: "The one great point the Jenkins followers make, about the only one they have a shadow of a chance to make, is the greater ease with which gold can be burnished into a cavity to form a matrix, but the greater ease and safety with which a platinum matrix can be handled afterward more than counterbalances both in time saved and in accuracy of results obtained. I know of no cavity in which gold could be burnished that platinum could not be burnished better, and I can call to mind several in which, if gold were used, it would have been so distorted in removal from cavity as to be useless as a matrix."

Further on Dr. Reeves says, "I will burnish a matrix from start to finish in from fifteen to twenty minutes."

After the matrix is obtained there would seem to be little opportunity for argument regarding what body should best be used. The principles of building up an inlay in layers as first worked out and demonstrated by Dr. Reeves, seems so much in advance in its results of anything I have seen in similar cases, by use of the low-fusing body, that I would consider it a practical waste of time at least to give any special attention to the low-fusing porcelains.

But to return for a moment to the principal point I am especially anxious to emphasize at this time, I wish to say that the busy dentist should use porcelain whenever it is indicated and devote as many sittings to each operation as the conditions demand. Make yourself master of every situation and the appreciation from your patients that inevitably follows will more than repay for the efforts made.

Remember this, your patients will have confidence in your efforts in direct proportion as you show interest in their welfare. When you have developed that degree of skill in the manipulation of this material to which you aspire, your fees will be in proportion to the skill displayed and the results obtained.

DISCUSSION.

Dr. Hoff: The theme of Dr. Honey's paper strikes me as a very happy one at this time. It appeals to me with considerable force as I am one of the objectors to this work because of the time consumed by it. It probably is true that when I shall have done more of it and become more proficient I shall also become an enthusiast. There is one fact that appeals strongly to me because of this presentation, and that is that if such busy and capable men as Dr. Honey can find time, and esteem it worth their while to work up this subject, it is about time for me to swing into line. There are always a certain number of men in our profession who take up with the passing shows and climb into the band wagons and make a lot of noise until the procession gradually recedes from view. But this work seems to be making its appeal to the more thoughtful and practical men in our profession, and it is one of the hopeful indications that it has come to stay.

If, as the essayist and the distinguished gentlemen he quotes, assure us it is true that recurrent decay is less frequent about porcelain fillings than any other material now in use, then it becomes incumbent as a moral duty upon all of us that we get busy at once in this kind of work, for it is our duty to make use of every facility that a kind Providence puts into our hands for the alleviation and prevention of human suffering. There is existing in too large a measure for a profession the commercial aspect of our calling. We seem compelled to sacrifice our noblest principles in giving way to appeals for easy, cheap and painless services, from those who really have no right to demand such concessions, and who many times could be enlightened to their own benefit if we only would take the time and care to do it. It is not so much a question of whether it will be profitable to us in a pecuniary way or not, but the question which ought to most concern us, is it the best thing we can do for the welfare of our patient, that should decide us to undertake it regardless of time, trouble or expenses. Dr. Honey and others who have taken to this work report their patients, appreciation, and the results satisfactory from both the technical and artistic standpoints; and it is by such strong evidence that we are to grow and develop that high order of skill which shall enoble our lives and elevate the quality of the services our profession can render to the world. I thank Dr. Honey most cordially for presenting this aspect of this much-talked-of subject, as I believe it will arouse a new interest among us in this work which assuredly has come to stay, and to be one of the most approved methods of filling teeth.

Dr. H. M. Moorman: I am much pleased with the paper, but must take exceptions to the essayist's references to the low-fusing bodies and the gold matrix. I have done considerable inlay work it is true, most all of it with the low-fusing porcelains. I have tried the platinum matrix, and either I don't know how to manipulate it, perhaps I can manipulate the gold better, or it is not as easily manipulated, or I don't prepare my cavities properly. So far as getting artistic results are concerned I think I accomplish this object very well indeed. I approve of the Reeves method of building the colors in separate layers, and use the low-fusing, or Jenkins, bodies in accordance with that suggestion.

Dr. Honey: I would say that I do not wish it understood that I have been able to pick up this work from reading it in the journals. I have watched porcelain workers wherever I have had a chance to see them operate. I have perhaps gained much from watching Dr. Reeves and Dr. Raymond in their clinics given at our various society meeting during the last two years; but much can be learned from reading the subject, and best of all by actually doing the work in your own office and for your patients. Dr. Raymond made for a patient of mine an inlay in two hours and a half that I am confident I could not have made of gold in the same time. My first inlay in an approximal cavity was spoiled in setting by using undue force. My method now is to separate the teeth well with a Perry separator and insert my inlay and with a wooden wedge previously shaped, force the inlay gently to place by the wedge which is left in position until the cement has hardened. The separator should be loosened or removed if practicable as soon as the wedge is adjusted, by this means the pressure is equalized and does not come as a shock.

I have experienced no difficulty whatever in inducing my patients to allow me to insert porcelain in place of other materials, in fact they display unusual interest and are so appreciative that they do not hesitate to pay good fees for the service.

In regard to the difficulties Dr. Moorman speaks of, in working the platinum for a matrix, I would say that even a considerable tear in the platinum matrix does not disqualify it in cavities which are deep or large. The porcelain will flow over a considerable crack without passing through. In conforming the matrix to the cavity I use wet balls of cotton and crowd them into the cavity under considerable pressure. For holding the matrix in position while burnishing the edges, I find the wide rubber bands bought of the stationer are preferablk to strip of rubber dam, which is too thin and elastic. The getting of colors and handling of the furnace are important elements in the successful use of porcelain for inlays.

